# Spatial Allocation Method of Evacuation Guiders in Urban Open Public Spaces: A Case Study of Binjiang Green Space in Xuhui District, Shanghai, China

**DOI:** 10.3390/ijerph191912293

**Published:** 2022-09-27

**Authors:** Yanyan Niu, Jia Yu, Dawei Lu, Renwu Mu, Jiahong Wen

**Affiliations:** 1 School of Environmental and Geographical Science, Shanghai Normal University, Shanghai 200234, China; 2 Key Innovation Group of Digital Humanities Resource and Research, Shanghai Normal University, Shanghai 200234, China

**Keywords:** urban open public space, emergency evacuation, evacuation guider, Particle Swarm Optimization (PSO) algorithm, gradual covering model, responsibility area

## Abstract

Evacuation guiders play an important role when emergency events occur in urban open public spaces. Considering the shortcomings of the existing studies, an optimization method based on the Particle Swarm Optimization (PSO) algorithm and gradual covering model for spatial allocation of evacuation guiders in urban open public spaces is proposed. This method considers the impact of obstacles on intervisibility between guiders and evacuees, and the non-linear changing characteristics of the evacuation guiding quality based on the distances between guiders and evacuees to optimize the space allocation of evacuation guiders in urban open public spaces. Based on the emergency evacuation simulation, the evacuation efficiencies before and after the optimization of evacuation guider allocation can be compared to verify the validity of the proposed method. Furthermore, in order to improve the applicability of this method, the responsibility areas of the evacuation guiders are zoned according to different time periods. A case study of Binjiang Green Space in Xuhui District, Shanghai, China was conducted to demonstrate the feasibility of the proposed method. The results showed that the spatial allocation of evacuation guiders was highly correlated with the dynamic spatial change of evacuees. The reasonable spatial allocation optimization of evacuation guiders can effectively improve the emergency evacuation quality and reduce evacuation risks. The zoning of the evacuation guiders’ responsibility areas can help to clarify the responsibility area of each guider and provide a daily safety precaution scheme under a limited number of guiders. The method can provide detailed decision support for the security precaution of security staff and emergency evacuation management in urban open public spaces.

## 1. Introduction

In recent years, the occurrences of emergencies in urban open public spaces all over the world, such as crowd stampedes, explosions and terrorist attacks, have caused huge losses of people’s lives and properties. The security management in urban open public spaces is critical for the construction of harmonious societies [1]. Timely and effective emergency evacuation guiding is crucial to reduce or avoid casualties in urban open public spaces [2,3]. Moreover, urban open public spaces are open to the public all day long, such as public green spaces, pedestrian streets and city squares. They are prone to crowd aggregation due to the uncertainty and easy aggregative character of urban crowd flows, which requires more effective emergency evacuation guidance [4].

The evacuation guiders in urban open public spaces play important roles in emergency evacuation [5]. An evacuation guider refers to a security staff member who is familiar with the internal structure of an open public space, such as the evacuation exits, evacuation paths and other facilities, and can guide the evacuees to be evacuated as quickly as possible in the process of emergency evacuation [6]. In the research field of emergency evacuation, the guidance methods of evacuation guiders are generally divided into two types: static guidance and dynamic guidance. Static guidance means that the evacuation guiders convey information about the direction of evacuation to the evacuees without changing their position. However, in the dynamic guidance procedure, the evacuation guiders continually change their positions. Dynamic guidance can also be divided into two types. One is adsorption guidance, in which the evacuation guiders guide the surrounding evacuees to be evacuated together without conveying the evacuation direction information. The other is directional guidance, in which the evacuation guiders convey the evacuation direction information to the evacuees in the evacuation process, while they move toward the positions which have greater evacuation needs [7,8].

Compared with static guidance, dynamic guidance has been studied more extensively in the field of emergency evacuation. For example, Rozo and Arellana (2019), Zhao (2016), Mohamed and Basma (2019) and other scholars simulated the evacuation guiding process of evacuation guiders based on the multi-agent technology and social force model [9,10,11]. Moreover, on the basis of the cellular automata model, many scholars have simulated the evacuation process of crowd flows by considering various factors, such as hazard source, exits and conformity behaviors [12,13,14,15]. Bayram (2016) and Aea et al. (2019) studied the importance of reasonable planning of emergency evacuation routes in emergency evacuation [16,17]. Cui et al. (2008) studied the dynamic guided evacuation paths based on the Tabu search algorithm [18], and Abdelghany et al. (2014) set a reasonable guided path by an optimizing genetic algorithm [19]. The above studies focus more on the exploration of the evacuation guiders’ dynamic guidance process and guiding paths, but ignore the spatial allocation of the evacuation guiders at the initial stage of emergency evacuation.

When studying the role of evacuation guiders in public spaces, Li et al. (2006) found that the spatial allocation optimization of evacuation guiders in densely populated areas can effectively improve the quality of evacuation guidance. Meanwhile, they also preliminarily explored the influence of the number and locations of guiders on evacuation efficiency [6]. Some scholars also proved that reasonable spatial allocation of guiders can effectively shorten the emergency evacuation time [20,21]. For optimizing the spatial allocation of evacuation guiders, the main factors, such as the number of guiders, the guiding range of guiders and the spatial location of guiders, are generally considered, which have a high similarity to the spatial allocation of public facilities. Currently, the Lagrangian relaxation algorithm [22], greed algorithm [23], genetic algorithm [24,25], simulated annealing algorithm [26], ant colony algorithm [27,28], Particle Swarm Optimization (PSO) algorithm [29,30,31,32,33] and other heuristic algorithms as well as the multi-objective evolutionary algorithm [34] are commonly used in the research of public facility allocation problems. Heuristic algorithms can integrate qualitative and quantitative problems well and meet the needs of multi-objective decision making. Taking Beijing subway station as an example, Zhou et al. (2018) established an optimization model of station evacuation guiding which considered time, number of guiders and safety cost. The model improved the maximum covering model to optimize the number and initial positions of the guiders [35]. Cui et al. (2008) conducted the spatial allocation of static guiders according to the principle of the maximum covering problem in public service site selection. Their case study of simulating emergency evacuation in a supermarket demonstrated that the reasonable spatial allocation of guiders was conducive to improving the efficiency of emergency evacuation [36]. Judging from the above studies, it could be found that the reasonable spatial allocation of guiders is of great significance for effective emergency evacuation in urban open public spaces if emergency events occur. However, at present, most studies do not consider the influence of obstacles on the guider’s field of vision, which in turn affects the guider’s guiding range. Furthermore, in the actual guiding process, the guiding quality of the guiders will be weakened with the increase in the distance between guiders and evacuees, but this is less considered in the current study. Thirdly, the crowd distribution in an open public space is always dynamically changed, but the existing spatial allocation methods of evacuation guiders mostly neglect the spatiotemporal crowd distribution and do not provide relative responsibility recommendation for the security staff in urban open public spaces.

To solve the above problems, this paper proposes a spatial allocation method of evacuation guiders in urban public spaces based on the PSO algorithm and gradual covering model. The method considers the influence of obstacles on intervisibility between guiders and evacuees, the non-linear changing characteristics of the evacuation guiding quality based on the distances between guiders and evacuees and the guiding threshold (the maximum number of evacuees that each guider can guide). The evacuation efficiencies before and after the spatial allocation optimization of the evacuation guiders are compared based on the emergency evacuation simulation to verify the effectiveness of the spatial allocation results. Furthermore, in order to clarify the responsibilities of the evacuation guiders, the guiders’ responsibility areas are zoned by the Thiessen polygon method. The new proposed method can improve the rationality and feasibility of the spatial allocation of the evacuation guiders and the evacuation guiding process. Meanwhile, it also has applicability for the daily safety protection work of urban public spaces, which can improve the emergency management of urban public spaces.

**Figure 1:**
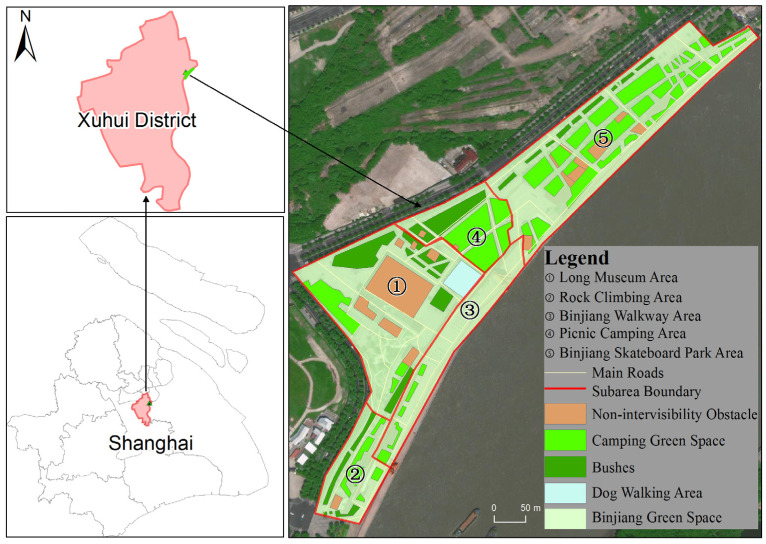
The study area in Binjiang Green Space, Xuhui District, Shanghai, China.

**Figure 2:**
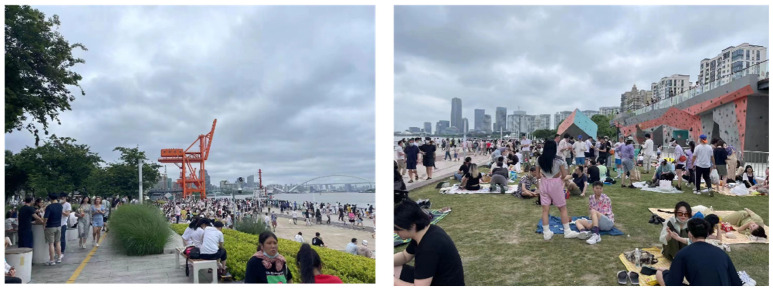
The photos of the evacuation points in Binjiang Green Space, Xuhui District, Shanghai, China on 5 May 2021.

A case study of Binjiang Green Space in Xuhui District, Shanghai, China was conducted to demonstrate the feasibility and effectiveness of the proposed method. Located near Ruining Road, Binjiang Green Space, Xuhui District, Shanghai, China is adjacent to the Huangpu River (Figure 1). It is a typical urban open public space, which is open for free public use throughout the day. We selected the main region of the Binjiang Green Space as the study area, which has the total area of about 9.4 km^2^. The region includes crowd gathering places such as the Long Museum Area, the Rock Climbing Area, the Binjiang Skateboard Park Area and other places where people gather in the daytime. It also includes a large picnic green space and basketball ground which have remarkable crowd gathering situations (Figure 2). By the field surveys, it was learned that the highest crowd flow at a certain moment in the region could reach more than 2000 people (15:00–17:00) during the holidays of the COVID-19 epidemic period. It can be estimated that the crowd number in the region will be much more than 2000 people during the holidays in normal times. For the safety management during the holidays and festivals, the number of evacuation guiders (security staff) arranged in this region is relatively insufficient. In other words, there is an inadequate supply of guidance services in this region during the peak crowd period. Therefore, it is essential to reasonably arrange the evacuation guiders in this region and optimize the spatial allocation of the evacuation guiders. The reasonable and effective evacuation guider allocation strategy can improve the effectiveness of evacuation guiding and provide technical support and decision guidance for emergency evacuation management in the urban open public space.

## 2. Methodology

**Figure 3:**
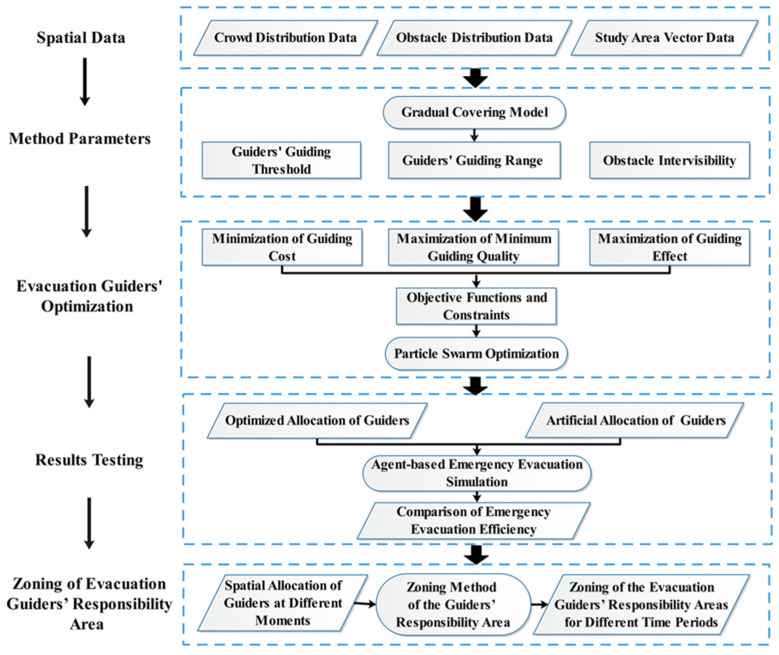
Schematic of the solution procedure for the allocation of evacuation guiders in urban open public spaces.

The framework of this study is illustrated in Figure 3. The spatial data include crowd distribution data, obstacle distribution data and study area vector data. The method parameters include guiders’ guiding threshold (maximum number of evacuees that each guider can guide), guiders’ guiding range and obstacle intervisibility. The guiders’ guiding range is set using the gradual covering model. The optimization objectives include minimization of guiding cost (minimizing the number of guiders), maximization of minimum guiding quality and maximization of guiding effect (maximizing the total guiding quality received by the evacuees), and using the PSO algorithm to optimize the allocation of evacuation guiders according to the objective functions and constraints. In order to verify the validity of the optimization results, the evacuation efficiency of the optimized allocation scenario is compared with the artificial allocation scenario by using agent-based emergency evacuation simulation. Considering the dynamic evacuee (crowd) distribution at different moments, based on the optimization result of spatial allocation of guiders at each moment, the zoning method of the guiders’ responsibility areas is built to clarify the responsibility area of each guider.

### 2.1. Spatial Allocation Optimization of Evacuation Guiders in Urban Open Public Spaces

Many evacuation studies defined the activity space of evacuees on the road network, and arranged evacuation guiders according to the risk value of road network nodes [37]. However, due to the particularity of urban open public spaces, the activity space of evacuees in this study is defined on a more microscopic scale, i.e., all geographic features are of the polygon type. Generally speaking, when conducting the spatial allocation of evacuation guiders, it is necessary to clarify the number of guiders, guiding objects, guiding positions, guiding range and other information, while considering the obstacle intervisibility at the same time. In this case, multiple objectives need to be satisfied, such as maximization of guiding effect, minimization of guiding cost, maximization of the minimum guiding quality and whether each evacuee can be guided. To resolve this multi-objective problem, this study integratively utilized the PSO algorithm and gradual covering model to build the new spatial allocation optimization method of evacuation guiders in urban open public spaces.

The PSO algorithm is a commonly used optimal location algorithm, which has good search efficiency and practicability in different studies worldwide [38]. PSO simulates the foraging scene of birds to find the optimal solution by multiple iterative comparison. A group of particles with specific speeds, positions and fitness values are initialized in the feasible solution space to represent the potential feasible solution set of the optimization problems. Moreover, in this paper, each particle represents a potential feasible solution to the spatial allocation of evacuation guiders. By comparing the fitness values of each feasible solution, the individual extreme value and the group extreme value are found. In each iteration, the individual and group extreme values of the particles are updated, and the velocity and position of each particle are updated. The updating equations are as follows:

(1) $${V\;}_{\mathrm{id}}^{t+1}{=w\;\times \;V\;}_{\mathrm{id}}^{t}{+c}_{1}r_{1\;}\left({P\;}_{\mathrm{id}}^{t}{\;-\;X}_{\;\mathrm{id}}^{\;t}\right){+c}_{2\;}r_{2}\;\left(P_{\;\mathrm{td}}^{\;t}{\;-\;X\;}_{\mathrm{id}}^{\;t}\right),$$

(2) $${w=w}_{1}-\;v\times \frac{{\;w}_{1}{\;-\;w}_{2\;}}{u},$$

(3) $${X\;}_{\mathrm{id}}^{k+1\;}{=X\;}_{\mathrm{id}\;}^{k}{+V\;}_{\mathrm{id}}^{k+1}.$$

(a) In Equations (1)–(3), ${\;V\;}_{\mathrm{id}}^{t}$ represents the *t-th* velocity of the *d*-dimension of the *i-th* particle.

(b) Equation (2) is a linear decreasing function of weight specially introduced to balance the global and local optimization performance. *w* is a non-negative inertia factor, which representing the ability of the particle to inherit the speed in the previous iteration. The value of *w* affects the global and local optimization ability. *w*_1_ represents the maximum value of *w*. *w*_2_ represents the minimum value of *w*. *u* represents the maximum number of iterations, and *v* represents the current number of iterations.

(c) ${P\;}_{\mathrm{id}}^{t}$ and $P_{\;\mathrm{td}}^{\;t}$ represent the current individual extreme value and group extreme value of the d-dimension of the *i-th* particle, respectively. *c*_1_ and *c*_2_ are the individual and social learning factors which have values in the range of [0, 1]. *r*_1_ and *r*_2_ are two random numbers which also have values in the range of [0, 1], and ${X\;}_{\mathrm{id}\;}^{k}$ is the current position of the *d*-dimension of the *i-th* particle. $\left({P\;}_{\mathrm{id}}^{t}{\;-\;X}_{\;\mathrm{id}}^{\;t}\right)$ is the gap between the current value of the particle and its individual extreme value in the *d*-dimension, $\left(P_{\;\mathrm{td}}^{\;t}{\;-\;X\;}_{\mathrm{id}}^{\;t}\right)$ is the gap between the current value of the particle and group extreme value of the particles. Previous studies found that when *w*_1_ = 0.9, *w*_2_ = 0.4 and *c*_1_ = *c*_2_ = 1.49, the particles can identify the approximate location of the optimal feasible solution faster, better balance the global and local search ability of the particles, speed up the convergence speed and improve the performance of the PSO algorithm [39,40]. In this paper, *w*_1_, *w*_2_, *c*_1_ and *c*_2_ are assigned the values of 0.9, 0.4, 1.49 and 1.49, respectively.

**Figure 4:**
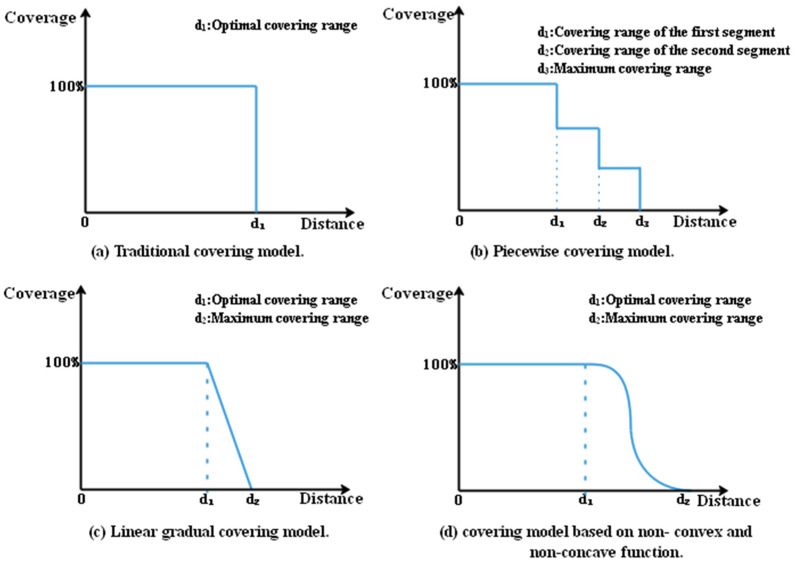
Schematic diagram of different covering models.

In the process of solving the spatial allocation problem of evacuation guiders using the PSO, the guiding effect of a guider to an evacuee is related to the distance between them. Therefore, it is necessary to select a suitable model to represent the guiding range and the guiding service quality received by evacuees. The covering model is selected to define the guiding range in this paper. The traditional covering model (Figure 4a) sets strict covering standards, which stipulate that the demand points within the covering range (*d*_1_) can obtain 100% service, while the demand points outside the service radius do not provide service. This strict dichotomy differs from the actual situation. Church and Roberts (1983) proposed the concept of a gradual covering model [41], which breaks the strict dichotomy of the traditional covering model. Then, with intensive studies, different improved models, such as the piecewise covering model, linear gradual covering model and covering model based on non-convex and non-concave functions, were gradually formed [42,43]. However, the piecewise covering model (Figure 4b) has discreteness, which is inconsistent with the continuous change in research objects in practical application. The linear gradual covering model (Figure 4c) considers the continuity of real objects, while its linear decreasing change struggles to express the complex research situation in reality. In contrast, the covering model based on non-convex and non-concave functions (Figure 4d) not only considers the continuous change in research objects and the complexity of the real situation, but can also better simulate the relationship between the distance and the service covering levels in real life. Therefore, this paper adopts the covering model based on non-convex and non-concave functions, i.e., the gradual covering model, to study the guiding range of the guiders. The specific equation is as follows:

(4) $$\;f\;\left(d_{\mathrm{ij}}\right)=\left\{\;\begin{array}{c} 1,\;0\leq {\;d}_{\mathrm{ij}\;}\leq {\;d}_{1}\\ \frac{1}{2}+\frac{1}{2}\;\cos\;\left(\frac{\pi }{d_{1}\;{-\;d}_{2}}\left(d_{\mathrm{ij}}\;-\;\frac{{\;d}_{1}{+d}_{2}}{2}\right)+\frac{\pi }{2}\right){,\;d}_{1}{\;<\;d}_{\mathrm{ij}}\;\leq {\;d}_{2}\\ {0,\;d}_{\mathrm{ij}\;}{>\;d}_{2} \end{array}\right..$$

In Equation (4), $f\;\left(d_{\mathrm{ij}}\right)$ represents the guiding service quality provided by the service point (the position of a guider) *j* to the demand point (the position of an evacuee) *i*, *d*_1_ represents the optimal guiding range and *d*_2_ represents the maximum guiding range. If the distance between *i* and *j* is within the optimal covering range *d*_1_, the demand point *i* can obtain 100% service from the service point *j*. When the distance between *i* and *j* is greater than the maximum covering range *d*_2_, service point *j* will not provide service to demand point *i*.

Integrating the PSO algorithm and the gradual covering model mentioned above, the multi-objective spatial allocation method of evacuation guiders in urban open public spaces is built. The method assumes that: (1) each evacuee must establish a guiding relationship with a guider. (2) After receiving the evacuation warning message, all the evacuation guiders clearly understand their guiding range and all information of the urban open public space, having the ability to conduct the evacuation guiding for the evacuees. (3) The guiding of the evacuation guiders is limited by the guiding threshold and the guiding range. The evacuation guiders do not return after leaving the guiding space. The equations are as follows:

(5) $$\max\frac{\sum_{\;i=1}^{\;n}\sum_{\;j=1}^{\;m}{\;f\;(d}_{\mathrm{ij}}{)\;S}_{\mathrm{ij}\;}L_{\mathrm{ij}}}{n\;\times \;\sum_{\;j=1}^{\;m}Z_{j}},$$

*max Q.* (6)

Subject to:

(7) $$\sum_{j=1}^{m}L_{\mathrm{ij}}=1,\forall \;i\in \;I,$$

(8) $$\sum_{i=1}^{n}L_{\mathrm{ij}}\;\leq \;K,\forall \;j\in \;J\;,$$

(9) $$\sum_{j=1}^{m}f\;\left(d_{\mathrm{ij}}\right)\;L_{\mathrm{ij}}\;\geq \;Q\;,\forall \;i\in \;I\;,\forall \;j\in \;J\;,$$

(10) $$Z_{j\;}=\left\{\begin{array}{c} 0,\;j\;\mathrm{is}\;\mathrm{selected}\;\mathrm{as}\;\mathrm{the}\;\mathrm{guider}\\ 1,\;j\;\mathrm{is}\;\mathrm{not}\;\mathrm{selected}\;\mathrm{as}\;\mathrm{the}\;\mathrm{guider} \end{array}\right.,$$

(11) $$L_{\mathrm{ij}}=\left\{\begin{array}{c} 0,\;i\;\mathrm{is}\;\mathrm{guided}\;\mathrm{by}\;j\\ 1,\;i\;\mathrm{is}\;\mathrm{not}\;\mathrm{guided}\;\mathrm{by}\;j \end{array}\right.,$$

(12) $$S_{\mathrm{ij}\;}=\left\{\begin{array}{c} 0,\;\mathrm{there}\;\mathrm{is}\;\mathrm{no}\;\mathrm{intervisibility}\;\mathrm{between}\;i\;\mathrm{and}\;j\\ 1,\;\mathrm{there}\;\mathrm{is}\;\mathrm{intervisibility}\;\mathrm{between}\;i\;\mathrm{and}\;j \end{array}\right..$$

*Q* is the minimum guiding quality obtained by evacuees. *n* and m represent the total number of evacuees and guiders, respectively. *I* and *J* are the sets of evacuees and guiders, respectively, and *K* represents the threshold of the number of evacuees guided by each guider. Equation (5) represents the maximization of the guiding effect. Equation (6) represents the maximization of minimum guiding quality. Equation (7) means that all evacuees can obtain guidance. Equation (8) describes that the number of evacuees guided by each guider does not exceed the threshold value *K*, and Equation (9) indicates that the guiding quality obtained by each evacuee is not less than the minimum quality *Q*. Equations (10)–(12) are used to explain the value range of the three decision variables in Equation (5).

### 2.2. Agent-Based Emergency Evacuation Simulation

In an agent-based model, an agent is an entity that can describe changes in itself and its surroundings, simulate people and other entities, perceive external information and share information with other agents to take corresponding actions. The agent-based model is characterized by autonomy, interactivity, responsiveness and proactivity [44]. In this paper, we use the agent-based model to simulate the emergency evacuation processes. The evacuation efficiencies before and after the optimization of evacuation guider allocation are compared to evaluate the effectiveness and practicality of the spatial allocation optimization method of evacuation guiders in urban open public spaces. We make some assumptions before the evacuation simulation: (1) evacuees are unfamiliar with the evacuation space and evacuation situation during the evacuation process, and need to be guided by an evacuation guider for effective evacuation. (2) Evacuees can only select one evacuation guider to follow. (3) When the movement target is identified, the evacuees will immediately go to it.

To conduct the evacuation simulations, it is necessary to construct the evacuation space, the evacuee agents, the evacuation guider agents and the behaviors of agents.

**Figure 5:**
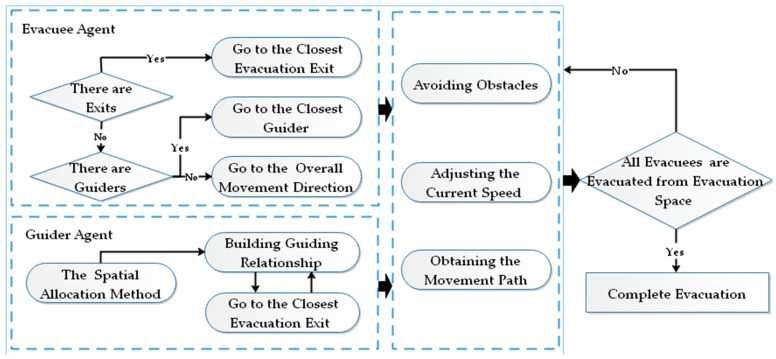
Flow chart of the evacuation behaviors of the agents (the evacuation guiders and evacuees).

(1) Evacuation space for the open public space: The environment modeling of the open public space is an important basis of the crowd evacuation simulation. In this paper, the buildings, lawns and bushes in the open public space need to be built. In the model of the evacuation environment, a navigation grid is set as the movement space of people.

(2) Attribute of the evacuation guider agent: The evacuation guiders play an important role in the process of emergency evacuation. The guider is responsible for guiding the orderly evacuation of the crowd in a public space when an evacuation warning is received. The attributes of an evacuation guider agent (Table 1) include the weight, height and the shoulder breadth of the guider, the initial position, the initial speed, the maximum speed, the current position, the current speed, the movement direction, the guiding target, the guiding path, etc. Existing studies show that there is a better evacuation effect when the speed of the evacuation guider is 75% of that of the surrounding evacuees [20]. Therefore, the maximum speed of the evacuation guider agent is set to 75% of the maximum speed of the evacuee agents in this paper.

(3) Attribute of the evacuee agent: The attributes of the evacuee agent include the initial position, the initial speed, the maximum speed, the current position, the current speed, the movement direction, the environmental visible radius, the position of the evacuation guider followed, etc. Some values of the attributes can be changed during the evacuation simulation (Table 2). Since evacuees are prone to panic and less familiar with the evacuation space in the process of emergency evacuation, their environmental visible radius is lower than that of the guiders, which is set to 80 m in this study.

(4) The behaviors of the agents (the evacuation guiders and evacuees) are as follows (Figure 5):

(a) The guiding relationships between the guiders and the evacuees are determined by the multi-objective spatial allocation method of evacuation guiders when there are evacuation guider agents but no evacuation exit in the surrounding environment of the evacuee agents.

(b) The evacuee will give priority to the closest evacuation exit to complete the evacuation when there are evacuation exits in the surrounding environment.

(c) When the evacuees fail to obtain effective evacuation information, they will follow the crowd, which causes crowd gathering. At this time, the movement direction of the evacuee agent tends to be the same as the overall movement direction of the surrounding crowd.

(d) In the evacuation process, the evacuation guiders select the closest evacuation exits as the guiding target, and the movement direction of agents (guiders and evacuees) changes in order to avoid obstacles.

(e) The current speed of an agent (guider or evacuee) is related to the surrounding crowd density. When the crowd density increases, the agent will reduce the movement speed. Lo et al. obtained the relationship model between the movement speed and the crowd density through 3000 simulation tests [45]. Referring to their study, the current movement speed of an agent is calculated as follows:

(13) $${\mathrm{Speed}}_{r\;}=\left\{\begin{array}{c} {\mathrm{Speed}}_{\max}\;,\;0\leq \;\rho \;\leq \;0.75\\ 0{.0412\;\rho \;}^{2}-\;0.59\;\rho +1.867,\;0.75\;<\;\rho \;\leq \;4.2\\ 0.1\;,\;4.2\;<\;\rho \end{array}\right.,$$

where *Speed_r_* (m/s) is the current movement speed of the agent, *Speed_max_* (m/s) is the maximum movement speed of the agent, ρ (person/m^2^) is the crowd density in the surrounding environment of the agent. When 0 ≤ ρ ≤ 0.75, *Speed_r_* = *Speed_max_*. In this situation, the crowd density does not affect the movement speed [46]. For higher crowd density situations, when ρ approaches 4.2, *Speed_r_* will reduce to about 0.12 m/s. In order to avoid the dead cycle in the evacuation simulation, a minimum movement speed is set to 0.1 m/s if ρ > 4.2 [45].

(f) Movement path of the agents: The evacuation space is spatially expressed by two-dimensional grids and the A-star algorithm is utilized as the pathfinding algorithm to plan the movement paths of the agent in the grid-based scene. The A-star algorithm is a common path heuristic search algorithm. Before utilizing the algorithm, the search area needs to be divided into polygonal grids, with the grid centroids as the path nodes. The path searching starts from the initial node and gradually searches for nodes to obtain the shortest path. In this study, the study area is divided into convex polygonal grids, and connecting lines between nodes are used as the paths between convex polygonal grids. A-star can be described as follows:

(14) $$F\left(n\right)=G\left(n\right)+H\left(n\right),$$

where *F*(*n*) is the shortest path from the initial node to the target node through the mid-node *n*, *G*(*n*) is the actual path cost from the initial node to the mid-node *n*, *H*(*n*) is the predicted path cost from the mid-node *n* to the target node. *H*(*n*) is usually determined by the Euclidean distance method, the Manhattan distance method or the diagonal distance method, and the Euclidean distance method is chosen in this paper [47,48].

### 2.3. Zoning Method of the Guiders’ Responsibility Areas

**Figure 6:**
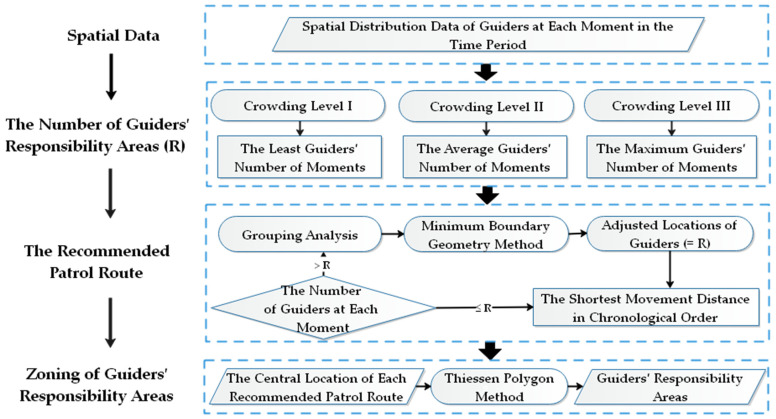
Flow chart of the zoning of guiders’ responsibility areas.

The number and locations of evacuation guiders need to be allocated based on the crowd density and crowdedness of the open public space. However, the crowd density in an open public space has strong dynamic and real-time characteristics. It is difficult to allocate the guiders according to the crowd distribution situation in real time. Therefore, the evacuation guiders’ responsibility areas can be zoned to meet the needs of daily safety precautions in open public spaces according to the crowd densities in certain time periods. Generally speaking, the areas which have a higher crowd density are more prone to safety accidents. Sufficient evacuation guiders need to be allocated in these areas [49]. According to the crowding perception of people and park use density index proposed by Baud-Bovy and Lawson (2004) and referring to the standard proposed by Fang et al. (2018) (they used 122 people/ha as the threshold between uncrowded and crowded perception in Gucun Park, Shanghai, China) [50,51], the crowding level classification standard of the open public space in this study is proposed in Table 3. According to this standard, different moments in a day can be divided into several time periods. The guiders’ responsibility area zoning is conducted for each time period respectively (Figure 6).

The zoning process is as follows:

(1) Determination of the number of the evacuation guiders’ responsibility areas (R) for each time period.

(15) $$R_{\;}=\left\{\begin{array}{c} \min\;\left(\;N_{i\;}{,\;N}_{i+1}\;{,\;\ldots ,\;N}_{j\;}\right),\;\mathrm{Crowding}\;\mathrm{level}\;\\ \frac{N_{i\;}{+N}_{i+1\;}{+\ldots +N}_{j}}{\;j\;-\;i}\;,\;\mathrm{Crowding}\;\mathrm{level}\;\mathrm{II}\\ \max\;\left({\;N}_{i}\;{,N}_{i+1\;}{,\ldots ,\;N}_{j}\;\right)\;,\;\mathrm{Crowding}\;\mathrm{level}\;\mathrm{II} \end{array}\right.$$

In Equation (15), *R* represents the number of evacuation guiders’ responsibility areas for a certain time period, *N_i_* and *N_j_* represent the first and last moments included in the time period, respectively. Equation (15) indicates that:

(a) When the crowding level is *I*, the smallest number of guiders in the time period from moment *i* to moment *j* is taken as the number of guiders’ responsibility areas in this time period.

(b) When the crowding level is *II*, the average number of guiders in the time period from moment *i* to moment *j* is taken as the number of guiders’ responsibility areas in this time period.

(c) When the crowding level is *III*, the maximum number of guiders in the time period from moment *i* to moment *j* is taken as the number of guiders’ responsibility areas in this time period.

(2) Determination of the recommended patrol route of the evacuation guiders for each time period.

(a) If the number of guiders of a moment is greater than *R*, the grouping analysis will be utilized to cluster the number of guiders at that moment into *R* classes, and find the central location of each class by the minimum boundary geometry method. The central locations are taken as the adjusted locations of the guiders at that moment. If the number of guiders of a moment is equal to or less than *R*, the number and locations of the guiders are not adjusted.

(b) In each time period, according to the principle of the shortest movement distance in chronological order, the locations of each guider in different moments are sequentially connected to generate the recommended patrol route of each guider.

(c) The central location of each guider’s recommended patrol route is obtained by the minimum boundary geometry method. These locations are taken as the central locations of the guiders’ responsibility areas in that time period.

(3) Zoning of the evacuation guiders’ responsibility areas.

According to the central locations of the guiders’ responsibility areas, the responsibility areas are zoned using the Thiessen polygon method [52]. The boundaries of the responsibility areas are further corrected according to the actual spatial pattern of the open public space. The responsibility areas and the recommended patrol routes of evacuation guiders can provide valuable decision support for daily safety protection in urban open public spaces.

## 3. Case Study

### 3.1. Data and Processing

After conducting the field surveys on several holidays, it was found that the changing and gathering trends of the crowd flows on different holidays were similar. The differences of the number of people at the same time on different holidays were not significant. The number differences were within 300 people. Therefore, this paper includes a typical holiday (Labor Day) as a case study.

**Figure 7:**
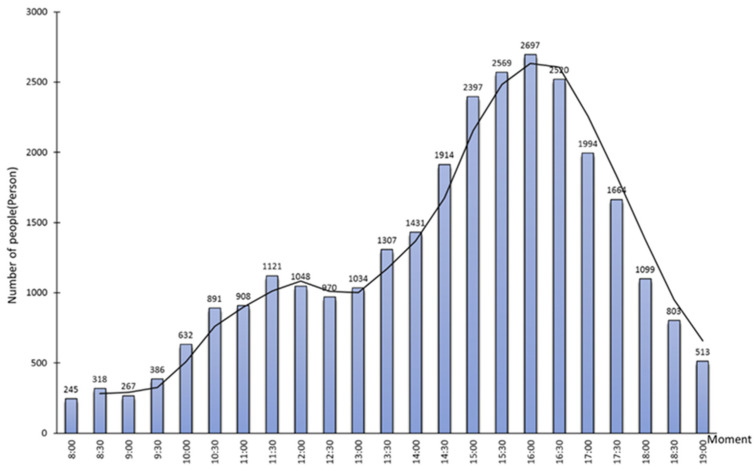
Changes in the number of people inside Binjiang Green Space, Xuhui District, Shanghai, China on 5 May 2021.

In this study, the crowd distribution data at 23 moments from 8:00 to 19:00 with the time interval of 30 min during Labor Day on 5 May 2021 (Labor Day is from 1 May to 5 May in China) were obtained by the field surveys. In the surveys, we divided the study area into five subareas (Figure 1), and each subarea has two responsible investigators. These investigators took 30 min as the time interval to obtain multi-media data by means of photo and video shooting, and interpreted them manually as spatial locations of the crowds. Finally, the spatiotemporal information of the crowd inside the study area was generated and stored in a spatial database. The changes in the number of people in the study area at different moments are shown in Figure 7.

The visual distance at which normal people can identify the type of scenery is between 150 m and 270 m [53]. Considering the influence of the crowding on the intervisibility and the effectiveness of evacuation guidance, the guiding range was set as follows: *d*_1_ = 100 m, *d*_2_ = 200 m. After several experiments, the population size *i* of the PSO was set to 100, the number of iterations *t* was 50 and the maximum number of evacuees guided by each evacuation guider *k* (the guiding threshold of guiders) was set to 100. Finally, by importing the data at each moment into the proposed allocation optimization method of evacuation guiders, the spatial allocation result of evacuation guiders at each moment can be obtained.

### 3.2. Allocation Optimization Results and Evaluation of Evacuation Efficiency

#### 3.2.1. Spatial Allocation Results of Evacuation Guiders at Different Moments

**Figure 8:**
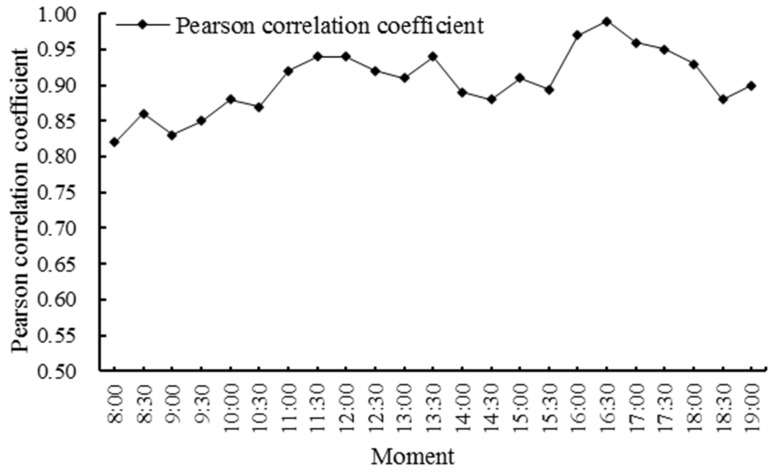
Changes in the Pearson correlation coefficient between the number of evacuation guiders and the number of evacuees at different moments on 5 May 2021.

**Figure 9:**
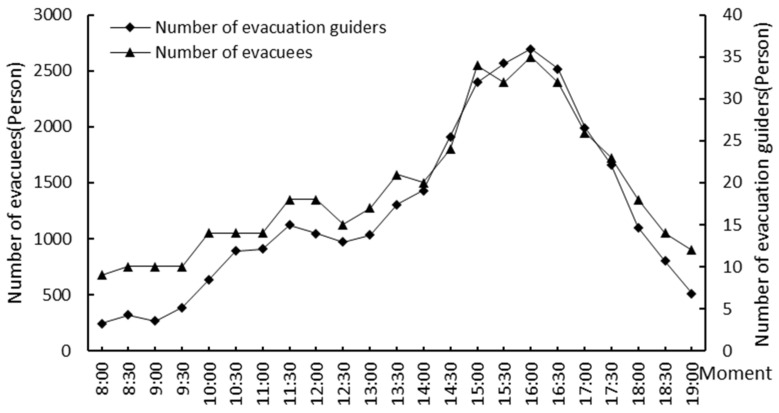
Changes in the number of evacuees and evacuation guiders at different moments on 5 May 2021.

We obtained the results of the spatial allocation of evacuation guiders at 23 moments in the study area. We counted the number of guiders and evacuees in five subareas (Figure 1) at different moments, and calculated the Pearson correlation coefficient between the number of guiders and the number evacuees at different moments (Figure 8). The Pearson correlation coefficient remained above 0.8, indicating a high positive correlation between the number of evacuation guiders and the number of evacuees, i.e., the more evacuees there are, the more evacuation guiders they need (Figure 9). Except for 12:00–13:00, the number of evacuation guiders and evacuees kept increasing continuously from 8:00–16:00. The number of evacuation guiders changed slightly from 8:00–9:30 because most of the people in the study area during this time period were residents nearby who came to do morning exercises, and there were not so many of these people. Moreover, from 14:30–16:00, the number of evacuation guiders and evacuees increased significantly, which was related to the fact that Binjiang Green Space provides a lot of afternoon camping and rest spaces for visitors. After 16:30, the numbers of evacuation guiders and evacuees in Binjiang Green Space continuously decreased, but the numbers were still more than those of 8:00–9:30.

**Figure 10:**
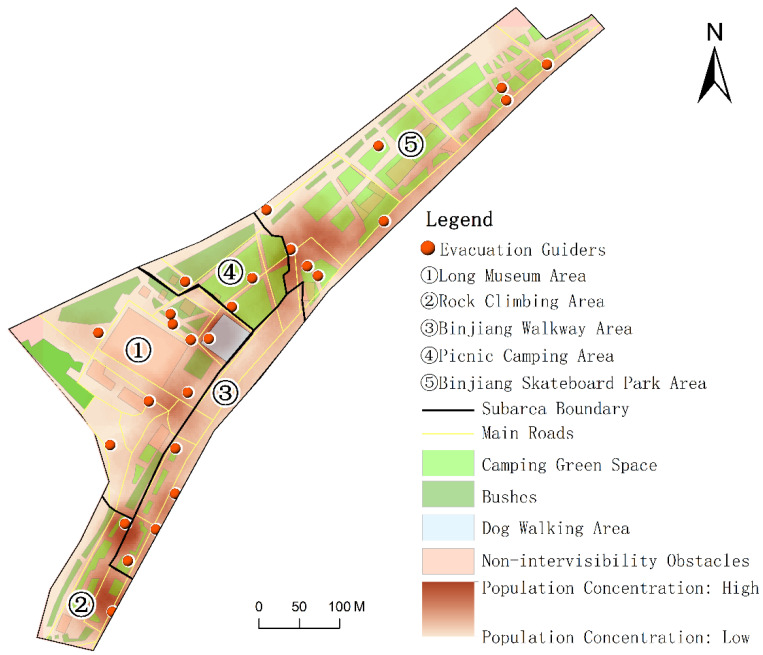
Spatial distribution of evacuees and spatial allocation of evacuation guiders at 17:00 on 5 May 2021.

In general, the evacuation guiders were mostly allocated in the areas where the evacuees were concentrated. For example, at 17:00, 26 evacuation guiders were needed, and about 4/5 of them were allocated in the areas where people gathered. Furthermore, there was a high concentration of evacuees in the Rock Climbing Area, near the Dog Walking Area, the southwest of the Binjiang Skateboard Park Area and the border between the Binjiang Skateboard Park Area and the Camping Green Space. Almost 50% of the evacuation guiders were allocated in these high-concentration areas (Figure 10).

**Figure 11:**
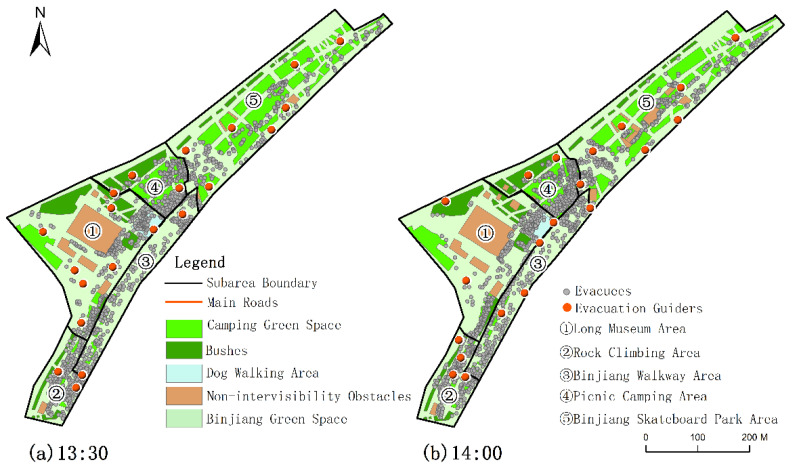
Spatial distribution of evacuees and spatial allocation of evacuation guiders at 13:30 and 14:00 on 5 May 2021.

With the number of evacuees increased, the number of the allocated evacuation guiders might decrease in some adjacent moments due to the impact of obstacles on intervisibility and crowd gathering degree. For example, the number of evacuees at 13:30 was less than that of 14:00, but the number of evacuation guiders was more than that of 14:00, because the distribution of evacuees at 14:00 was more concentrated than that of 13:30. Meanwhile, there were many obstacles in the northeast and southwest of the Long Museum Area, and more evacuees were among the obstacles in this area at 13:30 (Figure 11), which affected the intervisibility between the evacuees and the evacuation guiders. This situation reduced the guiding quality and increased the guiding cost.

#### 3.2.2. Evaluation of Evacuation Efficiency

In order to demonstrate the advantage of the spatial allocation method of evacuation guiders in urban open public spaces, analysis of the emergency evacuation efficiency is an effective measure to evaluate the performance of the method. Two scenarios were used for the comparison. The first is the “optimized allocation scenario”. Under this scenario, agent-based emergency evacuation simulation was conducted with the optimized locations of the evacuation guiders. The second scenario is the “artificial allocation scenario”. Under this scenario, the artificial evacuation guider allocation method was used, which simply arranged the evacuation guiders near the main evacuation exits and the centers of crowd gathering areas [20]. The agent-based emergency evacuation simulation was also conducted based on the artificial guider allocation result. The emergency evacuation efficiency of the “optimized allocation scenario” was compared with that of the “artificial allocation scenario”.

**Figure 12:**
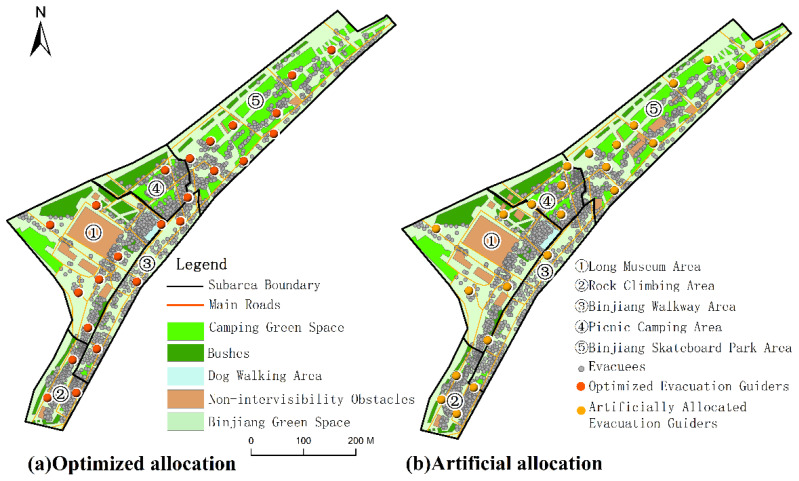
Optimized and artificial allocation results of evacuation guiders at 14:30 on 5 May 2021.

**Figure 13:**
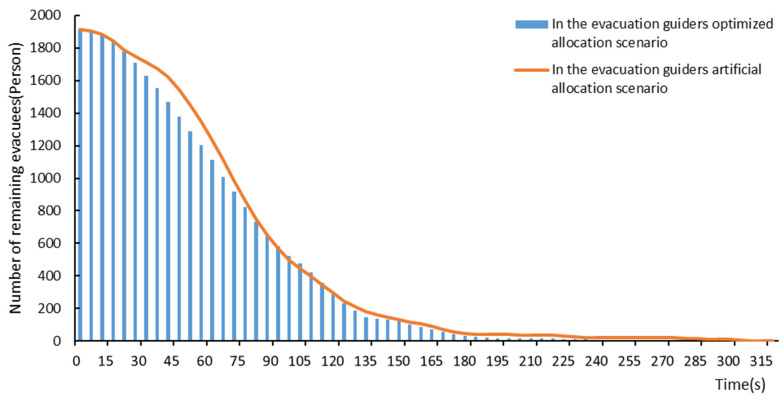
Comparison of evacuation efficiency between the “optimized allocation scenario” (the evacuation scenario using the proposed spatial allocation method of evacuation guiders) and “artificial allocation scenario” (the evacuation scenario using the artificial allocation method of evacuation guiders) at 14:30 on 5 May 2021.

Taking 14:30 as an example, there were 1914 evacuees in the study area at 14:30, and 24 evacuation guiders were required after the calculation of the proposed method. Thus, 24 evacuation guiders were also artificially allocated in the study area (Figure 12). On the basis of the two different scenarios, the evacuation procedures were simulated respectively. After the evacuation efficiency comparison, it could be found that the evacuation efficiency of the “optimized allocation scenario” was better than that of the “artificial allocation scenario” (Figure 13). In terms of the evacuation time, it took 255 s to complete the evacuation for all the evacuees at 14:30 under the “optimized allocation scenario”, while it took 315 s under the “artificial allocation scenario”. The optimized allocation of evacuation guiders reduced the evacuation time by 60 s. In terms of the number of remaining evacuees of different times, it could be found that the number of remaining evacuees in the middle evacuation period (from 90 s to 180 s) was similar for both the “optimized allocation scenario” and “artificial allocation scenario”, while the numbers of remaining evacuees in the early evacuation period (from 0 s to 90 s) and later evacuation period (from 180 s to 315 s) were more in the “artificial allocation scenario” than in the “optimized allocation scenario”. This revealed that the optimized allocation of evacuation guiders could improve the efficiency of emergency evacuation and significantly reduce the total evacuation time.

### 3.3. Zoning Results of Evacuation Guiders’ Responsibility Areas

In reality, it is difficult to allocate different numbers of evacuation guiders with different spatial allocations at different moments in the daily safety management of urban open public spaces in real time. Therefore, based on the allocations of the evacuation guiders at different moments and considering the crowding level and the continuity between the moments, the 23 moments at 14:30 on 5 May 2021 were divided into four time periods and the number of evacuation guiders’ responsibility areas in each time period was determined according to the zoning method of the evacuation guiders’ responsibility areas (Table 4). Then, the recommended patrol routes and guiders’ responsibility areas were generated for several time periods.

#### 3.3.1. The Recommended Patrol Routes of Evacuation Guiders

**Figure 14:**
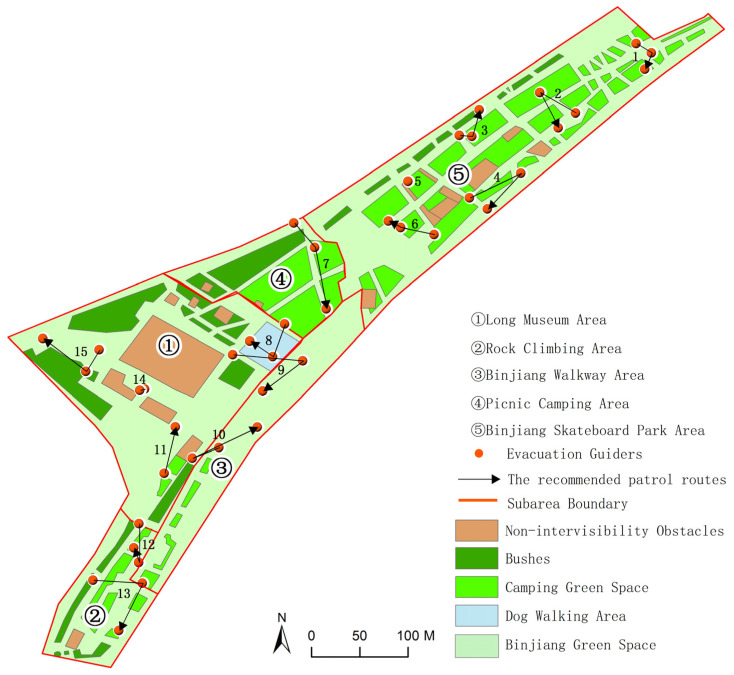
The recommended patrol routes of evacuation guiders from 18:00–19:00 on 5 May 2021.

In the same time period, due to the dynamic changes in the crowd distribution, different recommended patrol routes were generated. In order to better arrange the security work, considering the responsibility area and the length of the recommended patrol route, the guiders’ work modes were divided into two kinds: patrol mode and stand mode. Taking 18:00–19:00 as an instance, No. 5 guider had no patrol route, and undertook the stand-mode security work. No. 14 guider had the shortest patrol route, so this guider’s work mode was also set as the stand mode. No. 8 guider’s patrol route was inside the Dog Walking Area. This guider also took undertook the stand-mode work (Figure 14). Other guiders carried out the patrol-mode security work during the 18:00–19:00 time period.

Generally speaking, the longer the patrol route, the more energy the guider will consume, and the intensity of the patrol and guiding work will be increased. We used average patrol route length of the guiders to measure the intensity of the guiders’ work at different time periods. Although there was the highest number of evacuees from 14:30–17:30, because there was also the largest number of guiders, the work intensity of each guider was not high. The average patrol routes of the guiders from 10:00–14:00 and 18:00–19:00 were the longest and the shortest, respectively, and the number of guiders needed in these two time periods was similar. In order to deploy the work intensity more reasonably, the same group of security staff can be arranged as the guiders in these two time periods (Table 5). Considering the work intensity and the number of guiders, the guiders from 8:00–9:30 could join the security work from 14:30–17:30. In order to balance the work intensity, a shift system of the security work for the staff could be implemented in the study area.

#### 3.3.2. Zoning Results of the Evacuation Guiders’ Responsibility Areas

**Figure 15:**
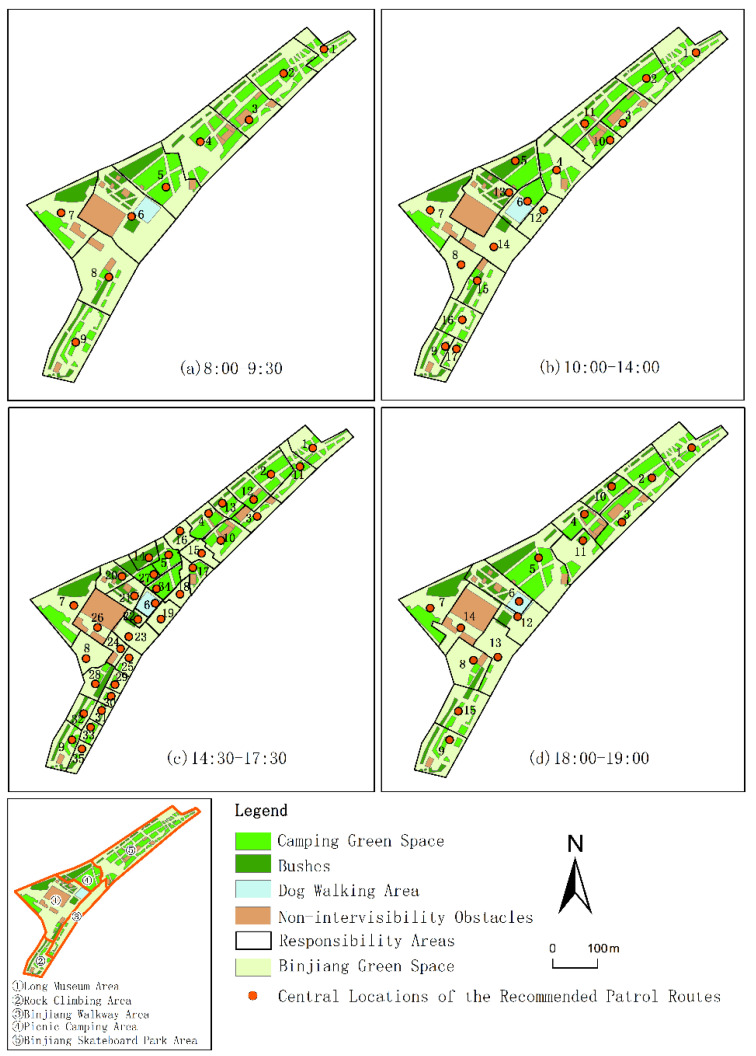
Zoning of evacuation guiders’ responsibility areas in different time periods.

According to the central locations of the recommended patrol routes in different time periods, the Thiessen polygon method was utilized to generate the responsibility areas. The boundaries of the responsibility areas were further corrected based on the spatial pattern of the study area (Figure 15). The number of responsibility areas from 8:00–9:30 to 14:30–17:30 continued to increase. From 18:00–19:00, the number of responsibility areas decreased significantly, only about 50% of the number from 14:30–17:30, but it was still more than the number from 8:00–9:30.

In terms of the spatial distribution of the responsibility areas, the evacuation guiders’ responsibility areas were basically zoned according to the northwest–southeast direction. With the increase in the number of responsibility areas, it appeared that the responsibility areas were zoned according to the northeast–southwest direction, which indicated that the responsibility areas were more meticulously zoned within the study area. Some responsibility areas had overlap at different time periods, such as the No.1–No.9 responsibility areas. Considering the reasonableness of the guiders’ allocation, the same guiders could be allocated in the No.1–No.9 responsibility areas. From 10:00–14:00, the responsibility areas were concentrated in the Picnic Camping Area, which was related to the picnic camping activities. Thus, the Picnic Camping Area required more security precaution during this time period. From 14:30–17:30, there were 35 responsibility areas, of which about 34% were located in the Picnic Camping Area and the Binjiang Walkway Area. This corresponded to the situation that about 35% of the total evacuees were in these areas in this time period. Moreover, the area of these responsibility areas only accounted for about 21% of the total area, which indicated that the crowd distribution in the Picnic Camping Area and the Binjiang Walkway Area was more aggregated than other regions from 14:30–17:30. These responsibility areas required more concentrated evacuation guiding.

#### 3.3.3. Discussion

**Figure 16:**
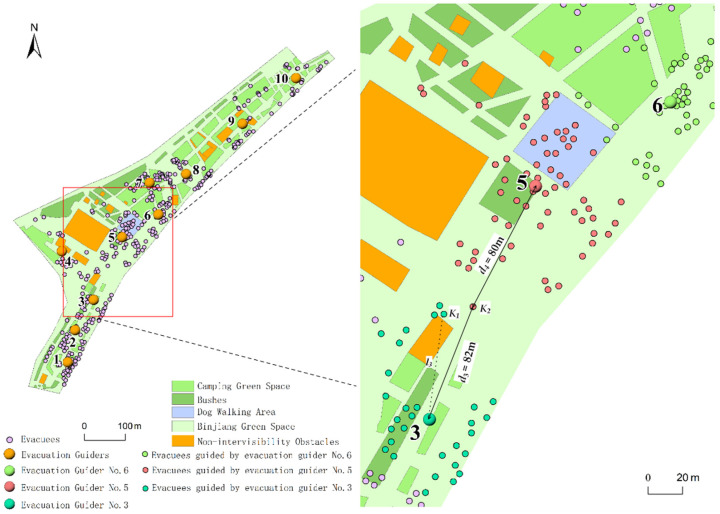
Schematic diagram of the allocation of the evacuation guiders, and the guiding relation between the guiders and the evacuees (based on k-means algorithm) at 9:30 on 5 May 2021.

**Figure 17:**
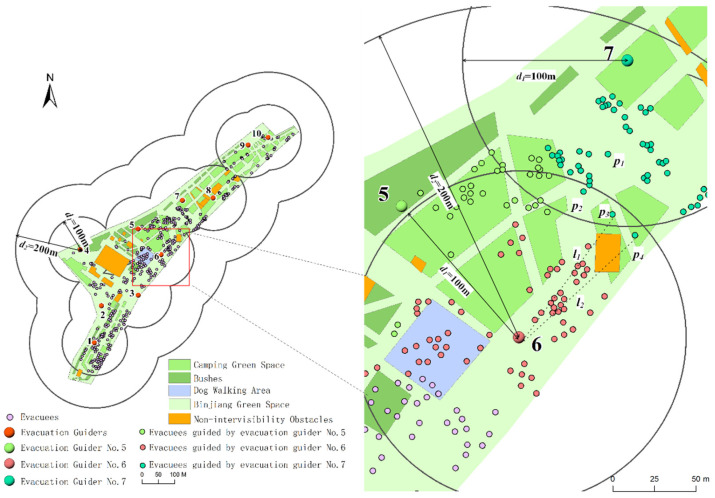
Schematic diagram of the allocation of the evacuation guiders, and the guiding relation between the guiders and the evacuees (based on the proposed method in this paper) at 9:30 on 5 May 2021.

The k-means algorithm [54], genetic algorithm [25] and maximal covering model [35] were mostly used in the existing studies to optimize the spatial allocation of evacuation guiders. The k-means algorithm obtains the spatial allocation of the guiders by clustering the locations of the evacuees, and does not take into account factors such as the intervisibility between the guiders and the evacuees, the guiding range of the guiders and the changing distance-based guiding quality characteristics of the guiders. In order to further explore the advantages of the proposed method in this paper, it was compared with the k-means algorithm. Taking the optimized allocation results of evacuation guiders at 9:30 on 5 May 2021 as an instance, a total of 10 evacuation guiders were needed for the study area. Figure 16 and Figure 17 illustrate the allocation results generated by the k-means algorithm and the proposed method in this study, respectively. In Figure 16, there is an obstacle between the No. 3 guider and *K*_1_ evacuee (*l*_3_), but the guiding relationship was still established. It did not conform to the actual situation because the obstacle would affect the intervisibility between the guider and the evacuee. As for the *K*_2_ evacuee, the distance between the No. 3 guider and *K*_2_ evacuee (*d*_3_) was 82 m and the distance between the No. 5 guider and *K*_2_ evacuee (*d*_4_) was 80 m. Although the value of *d*_3_ was approximate with that of *d*_4_, the guiding quality provided by the No. 3 guider for *K*_2_ evacuee was 0. *K*_2_ evacuee could only be guided by the No. 5 guider. Meanwhile, the No. 5 guider guided a large number of evacuees, but the No. 3 guider only guided a smaller number of evacuees. It was because the non-linear changing characteristics of the evacuation guiding quality and guiding threshold (the maximum number of evacuees that each guider can guide) were not considered. This made the optimized allocation result not conform to the evacuation guiding situation and not feasible in the real guiding process.

To comparatively illustrate the mechanism and feasibility of the guider allocation method in this paper, the No. 6 and No. 7 guiders were taken as examples (Figure 17). The *p*_1_ evacuee was in the *d*_1_–*d*_2_ (100–200 m) guiding range of the No. 6 guider and in the 0–*d*_1_ (0–100 m) guiding range of the No. 7 guider. The guiding qualities *q* (0 ≤ *q* ≤ 1) for the evacuee of the No. 6 and No. 7 guiders were 0.83 and 1, respectively. Therefore, the *p*_1_ evacuee chose to follow the No. 7 guider who provided the higher guiding quality. The *p*_2_ evacuee was in the intersection area of the 0–*d*_1_ guiding range of the No. 6 and No. 7 guiders. This evacuee chose to follow the No. 7 guider who guided fewer evacuees at that time. The *p*_3_ and *p*_4_ evacuees were located in the 0–*d*_1_ guiding range of the No. 6 guider. However, the intervisibility between these two evacuees and the No. 6 guider was limited by the obstacle (*l*_1_, *l*_2_). Therefore, the guiding quality provided by the No. 6 guider for the *p*_3_ and *p*_4_ evacuees was 0. Meanwhile, the *p*_3_ and *p*_4_ evacuees were in the 0–*d*_1_ and *d*_1_–*d*_2_ guiding range of the No. 7 guider, respectively, and the guiding qualities were 1 and 0.94. The *p*_3_ and *p*_4_ evacuees were finally guided by the No. 7 guider.

With the above comparative analysis, it is clear that because the proposed method takes into account different important factors (such as influence of obstacles on intervisibility between guiders and evacuees, the non-linear changing characteristics of the evacuation guiding quality based on the distances between guiders and evacuees and the guiding threshold), its allocation results better conform to the real evacuation guiding situation compared with the traditional allocation method (k-means algorithm).

Through the field surveys in the study area, it was known that there are only 8–10 security staff in the study area during normal times, which could satisfy the security work from 8:00–9:30 during holidays. However, from 10:00–14:00 and 18:00–19:00, the required number of security staff was double the available number of evacuation guiders, while the crowd from 14:30–17:30 needed about 3–4 times the available number of security staff. Therefore, the security department can appropriately deploy more security staff in the study area at different time periods of the holidays to reduce the security risk and promote safety precaution. For example, the security staff can be divided into four groups, and each group has eight to nine staff. One group can be allocated from 8:00–9:00. Two groups can be deployed from 9:30–14:00 and 18:00–19:00, and the four groups are all allocated from 14:30–17:30. In order to balance the work intensity of different groups, a suitable shift system can also be designed for these security staff.

After conducting the spatial allocation of evacuation guiders considering the impact of obstacles on intervisibility between guiders and evacuees, and the zoning of the guiders’ responsibility areas, the key precaution areas could be revealed. In the peak hours on the holiday, the southwestern part of the study area, including the Long Museum Area, the Rock Climbing Area and the Binjing Walkway Area, required key precautions. The area around the Long Museum especially needed more evacuation guiders due to the impact of many obstacles on the guiding range of the evacuation guiders. Therefore, the security department has to strengthen the security work in those areas, allocate sufficient security staff and make reasonable evacuation guiding plans. Moreover, the security department should regularly carry out security training and emergency drills for the security staff, monitor the dynamic distribution of the crowd in the open public space in real time and install warning signs in the areas with many obstacles. Integratively utilizing these measures and the spatial allocation optimization of the evacuation guiders, the security management capability of the urban open public space can be significantly improved.

## 4. Conclusions

This paper proposes a spatial allocation method of evacuation guiders in urban open public spaces based on the Particle Swarm Optimization algorithm and gradual covering model. The method has two advantages compared with traditional methods: (1) this method considers the influence of obstacles on intervisibility between the guiders and evacuees in evacuation guiding and sets the constraints on the guiders’ spatial allocation optimization; (2) the non-linear changing characteristics of the evacuation guiding quality based on the distances between guiders and evacuees are considered, which are mostly neglected in traditional methods. The gradual covering model is integrated in the method to set the non-linear changing characteristics. These two advantages can improve the rationality of the evacuation guiders’ spatial allocation and evacuation guiding process. Furthermore, to support the daily safety protection and emergency management of urban public spaces, the zoning method of the evacuation guiders’ responsibility areas is also proposed for different time periods, which improves the applicability of this method.

A case study of Binjiang Green Space in Xuhui District, Shanghai, China was conducted to show the feasibility and applicability of the method. After the spatial allocation of evacuation guiders for different moments in a day, it was found that the spatial distribution of the evacuees and the spatial allocation of evacuation guiders in the study area showed a high correlation. The effectiveness of the method was demonstrated by comparing the evacuation efficiencies before and after the optimization of evacuation guider allocation based on the agent-based emergency evacuation simulation. The simulation result revealed that the spatial allocation optimization of the evacuation guiders can significantly improve the evacuation efficiency in the evacuation guiding. According to the crowding levels in the open public space, the 23 moments in a day were divided into four time periods. For these time periods, the zoning of the guiders’ responsibility areas was conducted to provide the recommended patrol routes and clarify the responsibility areas of the guiders in the urban open public space. With the field surveys, it was found that the security staff in the study area could not meet the demand of security precaution work on holidays. The required number of staff and recommended deployment strategy were proposed in this study. With the case study, it is demonstrated that the proposed spatial allocation method of evacuation guiders can improve the guiding quality and effectiveness of emergency evacuation guidance. It can further provide useful decision support to reduce the risk in emergency evacuation and improve the security and emergency management capability in urban open public spaces.

The advantage of the proposed method compared with the traditional spatial allocation methods of evacuation guiders lies in three aspects: (1) this study considers the changing of the evacuation guiding quality based on the distance between the guiders and evacuees, and integratively utilizes the PSO algorithm and gradual covering model to optimize the space allocation of evacuation guiders in urban open public spaces. (2) The proposed method takes into account the impact of obstacles on the intervisibility between the evacuation guiders and evacuees, which makes the spatial allocation of the guiders and the guiding relationship establishment between the evacuation guiders and evacuees more feasible and realistic. (3) The spatiotemporal crowd number and distribution changes in the urban public space are taken into consideration in this study. The proposed method can be implemented as an application for mobile phones to support crowd evacuation decision-making in emergency situations and improve efficiency of emergency management in open public spaces. The evacuation guiders’ responsibility areas are zoned for different time periods in a day, which helps to clarify the daily security responsibility of each guider (security staff) and provide a detailed safety precaution scheme for the emergency management of urban open public spaces.

The proposed method in this paper will be further improved in future studies, including the following aspects: (1) exploring the impact of different feature types (steps, lawn, bushes, etc.) on evacuation guiding quality and efficiency. (2) Considering the crowd and traffic situation outside the urban open public space and further optimizing the spatial allocation and guiding routes of the evacuation guiders.

## Figures and Tables

**Table 1:** Attributes of the evacuation guider agent.

Attributes	Value or Description
Initial speed (m/s)	0.975
Maximum speed (m/s)	1.05
Current speed (m/s)	The movement speed is determined according to Equation (13)
Shoulder breadth (m)	0.5
Weight (kg)	70
Height (m)	1.7
Environmental visible radius (m)	100
Initial position	The guider’s position before the evacuation simulation
Current position	Real-time guider’s position during the evacuation simulation
Movement direction	The direction of guider’s current position towards the guiding target
Guiding target	The evacuation exits closest to the guider
Guiding path	The guiding path of the evacuation guider generated by the A-star algorithm

**Table 2:** Attributes of the evacuee agent.

Attributes	Value or Description
Initial speed (m/s)	1.3
Maximum speed (m/s)	1.4
Shoulder breadth (m)	0.5
Current speed(m/s)	The movement speed is determined according to Equation (13)
Weight (kg)	70
Height (m)	1.7
Environmental visible radius (m)	80
Surrounding environment	The environmental visible covering range (a circular region) which takes 80 m as the radius and the location of the evacuee agent as the center
Initial position	The evacuee’s position before the evacuation simulation
Current position	Real-time evacuee’s position during the evacuation simulation
Movement direction	The overall movement direction towards the movement target
Movement target	Determined by the specific situation within the surrounding environment
Movement path	The movement path of the evacuee generated by the A-star algorithm
Position of the evacuation guider followed	Determined by the multi-objective spatial allocation method of evacuation guiders

**Table 3:** The crowding level classification standard of open public spaces.

Crowding Level (Number of Users/ha)		
Uncrowded (I)	Moderately Crowded (II)	Crowded (III)
0–40	40–122	>122

**Table 4:** Demand for evacuation guiders at different time periods in Binjiang Green Space.

Time Period	Crowding Level	Number of Evacuation Guiders Needed (Person)
8:00–9:30	I	9
10:00–14:00	II	17
14:30–17:30	III	35
18:00–19:00	II	15

**Table 5:** The average patrol distance of evacuation guiders at different time periods.

Time Period	8:00–9:30	10:00–14:00	14:30–17:30	18:00–19:00
**The average patrol route length (m)**	163	347	197	78
